# Feasibility Trial to Evaluate Tendon Stiffness Obtained from Shear Wave Elastography Imaging as a Biomarker of Aromatase Inhibitor-Induced Arthralgias

**DOI:** 10.3390/jcm11041067

**Published:** 2022-02-18

**Authors:** Jessica A. Martinez, Mihra S. Taljanovic, Andres A. Nuncio Zuniga, Betsy C. Wertheim, Denise J. Roe, Sima Ehsani, Sao Jiralerspong, Jennifer Segar, Pavani Chalasani

**Affiliations:** 1 The University of Arizona Cancer Center, Tucson, AZ 85719, USA; bwertheim@uacc.arizona.edu (B.C.W.); droe@arizona.edu (D.J.R.); simaehsani@arizona.edu (S.E.); sjiral@arizona.edu (S.J.); segar@arizona.edu (J.S.); pchalasani@uacc.arizona.edu (P.C.); 2 Department of Nutritional Sciences, University of Arizona, Tucson, AZ 85719, USA; 3 Department of Medical Imaging and Orthopaedic Surgery, University of Arizona, Tucson, AZ 85719, USA; mihrat@radiology.arizona.edu; 4 Department of Radiology, University of New Mexico, Albuquerque, NM 87131, USA; 5 Department of Biomedical Engineering, University of Arizona, Tucson, AZ 85719, USA; aanuncio@arizona.edu; 6 Department of Epidemiology and Biostatistics, University of Arizona, Tucson, AZ 85719, USA; 7 Department of Medicine, University of Arizona, Tucson, AZ 85719, USA

**Keywords:** aromatase inhibitors, breast cancer, shear wave elastography, ultrasound, joint pain, stiffness, arthralgia, aromatase inhibitor–induced arthralgia (AIA)

## Abstract

Aromatase inhibitor-induced arthralgia (AIA) comprises significant, activity-limiting musculoskeletal symptoms, including joint pain, myalgia, and joint stiffness. We conducted a prospective feasibility study in postmenopausal women diagnosed with early-stage (0–3) hormone receptor positive (HR+) breast cancer who were candidates for treatment with adjuvant AI therapy (*n* = 16). Tendons of the hands and wrists and the median nerve were imaged using gray-scale and power Doppler ultrasound (US) and US SWE. Arthralgia symptoms were evaluated using the Breast Cancer Prevention Trial (BCPT) Symptom Checklist musculoskeletal subscale (MS) and the Western Ontario and McMaster Universities Osteoarthritis Index (WOMAC) pain and stiffness subscales. At baseline, there were significant differences in the SW velocities of tendons between dominant and nondominant hands. Increased velocity in 2 of 6 tendons and the median nerve was associated with greater pain at baseline, whereas slower velocity of the extensor digitorum tendon (suggesting decreased stiffness) was associated with a higher WOMAC stiffness score. Increased SW velocity (suggestive of increased stiffness) at baseline in the abductor pollicis longus tendon was associated with a worsening of all three pain and stiffness measures by 6 months. Future studies should evaluate SWE scores related to AIA outcomes in a larger sample size.

## 1. Introduction

Aromatase inhibitor-induced arthralgia (AIA) comprises significant, activity-limiting musculoskeletal symptoms, including joint pain, myalgia, and joint stiffness [1]. AIA can also include carpal tunnel syndrome (CTS) [2,3], tenosynovitis, and muscle weakness [4,5]. Symptoms can affect the spine as well as large and small joints of the upper and lower extremities [6,7]. The median time to develop initial AIA symptoms is 6 weeks, with peak symptoms reported at 6 months [8]; however, some women experience worsening symptoms up to 1–2 years post-initiation of an aromatase inhibitor (AI) [9,10]. Extended adjuvant AI therapy (10 years) has been shown to improve disease-free survival when compared to placebo in women who completed 5 years of standard AI therapy [11]. Given the long duration of treatment, it is imperative to identify patients at risk for AIA in order to develop early interventions and improve quality of life and adherence to therapy. The development of biomarkers for AIA is an unmet clinical need which impacts a large patient population.

Previous studies have used Doppler ultrasound (US) to evaluate pathological changes of the tendons and joints associated with AIA. Our prior study suggested that women with AIA had non-significantly higher hyperemia and increased tenosynovial fluid relative to asymptomatic, age-matched controls using a Doppler US [12]. Similarly, Dizdar et al. showed that patients with AIA had significantly more fluid in the tendon sheaths and electrophysiologic findings of carpal tunnel syndrome relative to patients on AI without pain [4]. However, other studies using US and age-matched controls found no association between any US findings and AIA symptoms [13,14]. The inconsistency in relating US findings to AIA symptoms underscores the need for more sophisticated imaging techniques to better evaluate physiologic changes with AIA.

Shear wave elastography (SWE) is a novel imaging technique used to acquire a measure of tendon stiffness via shear acoustic waves of a focused ultrasonic beam [15]. In our prior case-control study, women with AIA had significantly faster SW velocities (suggesting stiffer tendons) than age-matched controls, determined by US SWE [12]. While this preliminary case-control study had a limited sample size, the finding of stiffer tendons with AIA was intriguing given that affected tendons have been shown to be softer than healthy normal tendons in other studies [16]. For example, Turkay et al. demonstrated that adult patients with de Quervain tenosynovitis had slower SW velocities (suggesting softer tendons) than healthy adults in the first extensor compartment of the hand [17]. Conversely, SWE studies of the median nerve in the assessment of carpal tunnel syndrome have shown increased SW velocities in patients relative to controls [18]. Other studies suggest that the relationship between pain and soft tissue stiffness on US SWE may vary by pathology. Breda et al. showed that patients with patellar tendinopathy had significantly increased SW velocities (suggesting increased tendon stiffness) compared to age-matched asymptomatic controls [19]. Hou et al. suggested that there was tendon softening on US SWE with rotator cuff disease [20]. Pan et al. showed a significant positive correlation between pain and increased SW velocities in patients with plantar fasciitis [21]. In the context of rheumatoid arthritis, muscle stiffness was not associated with muscle strength; however, that study did not evaluate tendon stiffness with imaging [22].

Here, in the context of an ongoing prospective study to evaluate the biomarkers of AIA in postmenopausal women, we evaluated tendon features of the hands and wrists using US SWE in a subset of patients. Tendon stiffness was evaluated at initiation and after 6 months of AI treatment. To our knowledge, this is the first study to use US SWE to associate tendon stiffness with AIA symptoms in breast cancer patients taking AI and to determine whether it is feasible to associate tendon stiffness at baseline with worsening AIA symptoms by 6 months.

## 2. Materials and Methods

### 2.1. Study Design

This study was embedded in an ongoing, single-arm, prospective clinical trial of patients with early-stage hormone receptor positive (HR+) breast cancer to evaluate and develop blood-based and imaging biomarkers of AIA. The trial was approved by our institutional review board and was conducted in accordance with the requirements of the provisions of the Declaration of Helsinki. All patients provided written informed consent. The trial was registered on clinicaltrials.gov (NCT03665077). All eligible patients completed their definitive treatment (surgery ± radiation) and were recruited at the time of their medical oncologist visit, prior to initiation of anastrozole as adjuvant therapy. For this sub-study, US SWE images were captured at the initiation of their AI (*n* = 16) and after 6 months (*n* = 9). The US SWE component of this study was originally planned for all participants; however, the US instrument was no longer available for continuation of SWE studies for reasons that were unrelated to the current study or the authors. Of the 16 patients with baseline SWE and questionnaire data, 14 had paired questionnaire data at 6 months.

### 2.2. Participants

Postmenopausal women diagnosed with early-stage (0–3) HR+ breast cancer who were candidates for treatment with adjuvant AI therapy were eligible for this trial. Exclusion criteria included prior breast cancer diagnosis, prior adjuvant or neo-adjuvant chemotherapy, prior endocrine therapy (AI or tamoxifen), history of rheumatoid arthritis or other autoimmune arthritis, daily NSAID use (other than baby aspirin), or any corticosteroids or immunosuppressive therapies.

### 2.3. BCPT-MS

The Breast Cancer Prevention Trial (BCPT) Symptom Checklist is a 42-item questionnaire validated in breast cancer survivors [23]. Here, we used the musculoskeletal subscale (BCPT-MS), which consists of the mean of responses to three questions addressing general aches and pains, joint pain, and muscle stiffness. The BCPT-MS subscale has been shown to be responsive to changes in AIA [24]. Scores range from 0–12, with higher scores representing worse symptoms.

### 2.4. WOMAC

The Western Ontario and McMaster Universities Osteoarthritis Index (WOMAC) is a 24-item instrument developed to assess pain, stiffness, and physical function in participants with osteoarthritis or AIA [25,26]. Here, we evaluated the pain (5 items) and stiffness (2 items) subscales using the 5-point Likert format (0 = none, 1 = mild, 2 = moderate, 3 = severe, and 4 = extreme). As discussed in Bellamy [25], for convenience and for comparison purposes to previous studies, total scores and each subscale were normalized to a range of 0–100.

### 2.5. Gray-Scale, Power Doppler, and Shear Wave Elastography (SWE) US Imaging and Scoring

All images were collected before and after the initiation of AI (baseline and 6 months). Real-time gray-scale and power Doppler US examination of the bilateral wrists were performed on a General Electric Logiq E9 machine using the 18–8 MHz linear hockey stick transducer as previously described [12]. Briefly, during gray-scale and power Doppler US examination, patients were seated with their hands resting on a small table placed between the examiner and the patient. All tendons and tendon sheaths were evaluated for the presence of a normal or increased synovial fluid complex on gray-scale evaluation and for the presence of active inflammation on power Doppler evaluation. Anatomical regions of interest included the abductor pollicis longus, extensor pollicis brevis, extensor digitorum tendon, extensor carpi ulnaris, flexor digitorum superficialis and flexor digitorum profundus tendons, and the median nerve.

SWE examinations of the bilateral wrists and scoring of these images were performed as previously described [12]. Briefly, a Siemens S3000 ACUSON US unit (Siemens Medical Systems) with a high-resolution (9–4 MHz) and 12-MHz linear transducers were used to optimize visualization of the examined regions and to accommodate depth. The tissue elasticity (degree of stiffness) was displayed on a color bar elastogram on the screen and expressed as SW velocities in m/s (scale: 0.5–20 m/s). A copious amount of US gel was also used to accommodate the depth. The anatomical regions of interest were collected along both long and short axes of the tendons/tendon sheaths.

To score SWE images, regions of interest (ROIs) were examined by a fellowship-trained musculoskeletal radiologist with more than 5 years of experience in US SWE imaging. ROIs contained the entire anatomical site of interest in each image. The B-mode images were co-registered with SW velocity color maps. The average SW velocity within each ROI was calculated using color map values and tabulated by a biomedical engineer. Each image was captured and scored in triplicate. The code to generate ROI’s is available on the public repository, GitHub.

### 2.6. Statistical Analysis

Baseline characteristics were summarized using the median and interquartile range (IQR) for continuous variables and proportions for categorical variables. Changes in symptom scores across time were tested using linear mixed-effects models, with time (interval since baseline) as a continuous variable, adjusted for the baseline symptom score, and clustered on the participant. Additional models further adjusted for age at baseline, BMI at baseline, and definitive therapy (mastectomy versus lumpectomy). For SWE data, the velocity at each image location (7 locations), axis (long or short), and side (dominant or nondominant) was measured in triplicate and reported as the mean ± SD or median (range). Changes in SW velocity across time were tested using linear mixed-effects models with time (interval since baseline) as a continuous variable, adjusted for transducer and baseline velocity (mean of the triplicate measures), clustered on the participant. The random-effect constant could not be reasonably estimated in 8 of the 28 models (one model for each location–axis–side combination), presumably due to the complexity of the model. Thus, a sensitivity analysis used the same models without clustering on the participant, and another sensitivity analysis did not adjust for the baseline velocity (but clustered on the participant). For the association between the baseline SW velocity and baseline symptoms, symptom scores were dichotomized into asymptomatic (score = 0) or symptomatic (score > 0) participants, and median velocities (mean of triplicate measures) were compared between these two groups using Wilcoxon rank-sum tests. Similar Wilcoxon rank-sum tests were used to compare the median baseline SWE velocity (mean of the triplicate measure) across groups of participants whose symptoms worsened or did not worsen between baseline and 6 months. No adjustments were made for multiple comparisons. Statistical analyses were conducted using Stata 17.0 (StataCorp, College Station, TX, USA).

## 3. Results

### 3.1. Participant Characteristics

The participants in the SWE subgroup were older adults with a median (IQR) age of 64.9 (63.5–71.5) years at enrollment (Table 1). Patients were enrolled shortly after diagnosis of their breast cancer. The Median (IQR) time between diagnosis and enrollment was 4.6 (3.3–6.3) months. Participants ranged from a healthy weight to obese, with a median (IQR) BMI of 25.9 (23.4–33.6) kg/m^2^, and the majority were non-Hispanic white (87.5%). There were 15 participants classified as right-hand dominant, and one was left-handed. For their definitive treatments, 75% had lumpectomy, and 62.5% had adjuvant radiation. There were three participants with stage 0 breast cancer, 11 with stage I, and two with stage II. All patients were still adherent to their AI at 6 months.

### 3.2. Gray-Scale and Power Doppler Ultrasound (US)

There was no increased power Doppler signal to suggest active inflammation at baseline or after 6 months in any participants on the Power Doppler interrogation. Additionally, all anatomical sites appeared normal using Grayscale US.

### 3.3. Baseline Shear Wave (SW) Velocity

Mean ± SD baseline SW velocities were compared between dominant versus nondominant sides in the long (Table 2a) and short (Table 2b) axes. The abductor pollicis longus had a significantly faster mean velocity, suggesting greater stiffness, on the dominant side (5.59 ± 2.46 m/s) relative to the non-dominant side (4.71 ± 2.14 m/s) in the long axis (*p* = 0.020), with no difference in the short axis.

**Figure 1:**
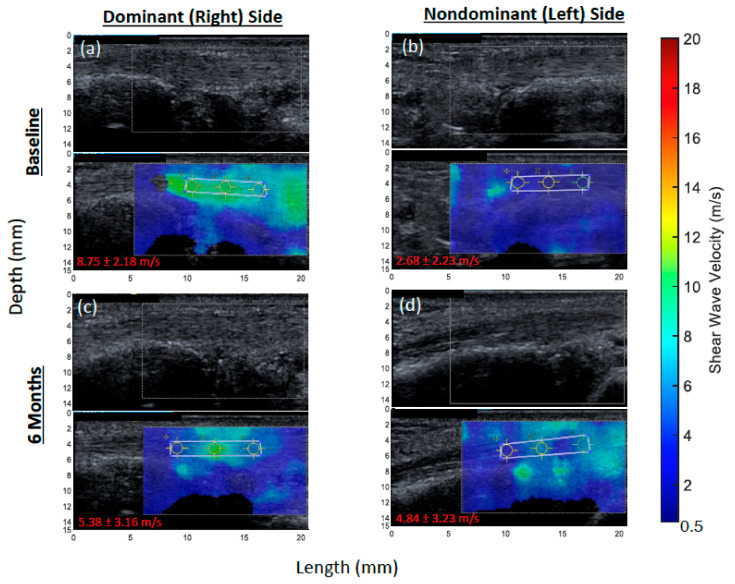
Representative shear wave elastography (SWE) images of the abductor pollicis longus (APL) tendon from a breast cancer patient initiating an aromatase inhibitor. Each elastogram (**bottom**) is displayed with its corresponding gray-scale image (**top**). Images were collected at the level of the wrist. At baseline, mean SW velocities were significantly faster on the dominant side relative to the nondominant in the long axis (*p* = 0.020). From baseline to 6 months, there was a significant reduction in SW velocity on the dominant side (*p* = 0.027) but not on the non-dominant side (*p* = 0.849). (**a**) APL tendon at baseline on dominant side. (**b**) APL tendon at baseline on non-dominant side. (**c**) APL tendon at 6 months on dominant side. (**d**) APL tendon at 6 months on non-dominant side. The shear wave velocity (mean ± standard deviation) was measured in meters per second (m/s) and is presented on the lower left corner of each image.

Differences between sides in the abductor pollicis longus at the baseline are illustrated for a representative patient (Figure 1a,b). There were no other differences in tendons by side in the long axis. There were differences in SW velocity by side in three of six tendons in the short axis: the extensor pollicis brevis (dominant: 4.72 ± 0.71 m/s versus nondominant: 4.29 ± 0.95 m/s; *p* = 0.007), flexor digitorum profundus (dominant: 4.53 ± 0.67 m/s versus nondominant: 5.18 ± 1.30 m/s; *p* < 0.001), and the flexor digitorum superficialis (dominant: 4.51 ± 0.82 m/s versus nondominant: 4.97 ± 1.43 m/s; *p* = 0.045). Additionally, the median nerve had a significantly slower mean velocity on the dominant side in the long axis (dominant: 5.56 ± 2.08 m/s versus nondominant: 6.18 ± 1.70 m/s; *p* = 0.020). Given these observed differences, all other results are stratified by side and axis.

### 3.4. Baseline Association between SW Velocity and Pain and Stiffness Scores

Median (IQR) SW velocities among symptomatic (score > 0) versus asymptomatic (score = 0) patients were compared for BCPT-MS (symptomatic, *n* = 12; asymptomatic, *n* = 4), WOMAC pain (symptomatic, *n* = 5; asymptomatic, *n* = 11), and WOMAC stiffness (symptomatic, *n* = 9; asymptomatic, *n* = 7). Patients that were symptomatic, as reported by the BCPT-MS, had a significantly faster median (range) SW velocity, suggesting greater stiffness, in the extensor carpi ulnaris (4.6 (3.3–4.9) versus 4.0 (2.7–4.3) m/s; *p* = 0.008 (nondominant side; short axis)) and the flexor digitorum profundus ((7.9 (4.8–9.9) versus 4.5 (2.7–6.8) m/s; *p* = 0.020 (dominant side; short axis)). Patients that were symptomatic using the BCPT-MS also had a faster median (range) SW velocity for the median nerve on the dominant side in both the long ((5.7 (4.2–9.2) versus 3.5 (2.5–5.8) m/s; *p* = 0.030) and short ((5.0 (4.0–7.7) versus 4.3 (3.8–4.6) m/s; *p* = 0.042) axes. The median SW velocity was not different in any other tendons for women with a score > 0 on the BCPT-MS at baseline on either side or in either axis. For women that were symptomatic on the WOMAC stiffness subscale, the extensor digitorum tendon had a significantly slower median (range) SW velocity ((4.8 (3.0–5.6) versus 5.4 (4.7–7.1) m/s; *p* = 0.009 (nondominant side; short axis)). No other tendons nor the median nerve were related to a score > 0 on the WOMAC stiffness subscale at baseline. No tendons nor the median nerve were related to a score > 0 on the WOMAC pain subscale at baseline.

### 3.5. Change in SW Velocity

Changes in SW velocity from baseline to 6 months of AI treatment were calculated in the 9 participants that had paired images (Table 3). The abductor pollicis longus showed a significant decrease in SW velocity (suggestive of tendon softening) on the dominant side in the long axis (β-coefficient = −0.024 m/s; *p* = 0.027) but no change on the non-dominant side or on either side in the short axis. Differences in the abductor pollicis longus from baseline to 6 months in the long axis on both sides are illustrated for a representative patient (Figure 1). The extensor carpi ulnaris showed a significant decrease in SW velocity on the dominant side in the long axis (β-coefficient = −0.033 m/s; *p* < 0.001) but an increase in the short axis (β-coefficient = 0.006 m/s; *p* = 0.040). The flexor digitorum superficialis had a significant decrease in SW velocity in the long axis on both the dominant (β-coefficient = −0.024 m/s; *p* = 0.014) and non-dominant (β-coefficient = −0.020 m/s; *p* = 0.038) sides. The median nerve had a significant increase in SW velocity on the dominant side in both the long (β-coefficient = 0.031 m/s; *p* = 0.002) and short (β-coefficient = 0.018 m/s; *p* = 0.009) axes, but it had a significant decrease in velocity on the non-dominant side in the long axis (β-coefficient = −0.021 m/s; *p* = 0.019).

### 3.6. Association between Baseline SW Velocity and Worse Pain and Stiffness Scores at 6 Months

We then sought to determine whether baseline SW velocity predicted a worsening of pain and stiffness scores from baseline to 6 months (BCPT-MS (*n* = 10 worsening, *n* = 4 non-worsening); WOMAC pain (*n* = 5 worsening, *n* = 9 non-worsening); WOMAC stiffness (*n* = 8 worsening, *n* = 6 non-worsening)). The abductor pollicis longus had significantly faster median (range) SW velocities at baseline in women that had a worse BCPT-MS at 6 months relative to women with a non-worsening score ((5.1 (4.1–8.9) m/s versus 3.8 (2.8–4.5) m/s; *p* = 0.024) and in women with a worse WOMAC stiffness score at 6 months ((5.1 (4.0–8.9) m/s versus 4.2 (2.8–8.4) m/s; *p* = 0.043), both in the long axis on the dominant side. Women that had worsening WOMAC pain scores also had a non-significantly faster median (range) SW velocity in the abductor pollicis longus at baseline relative to women with a non-worsening score ((8.4 (4.3–8.8) m/s versus 4.5 (2.8–8.9) m/s; *p* = 0.083; long axis, dominant side). The only other anatomical site associated with questionnaire scores at 6 months was the flexor digitorum superficialis, which had a significantly faster median SW velocity in women with a worsening WOMAC stiffness score relative to women with a non-worsening score ((6.7 (4.9–9.1) m/s versus 4.7 (4.1–6.0) m/s; *p* = 0.008 (long axis, dominant side)). There were no other significant differences in SW velocities at baseline for any anatomical sites with regard to worsening pain or stiffness scores at 6 months.

## 4. Discussion

This study was originally designed to determine whether baseline SW velocities (tendon stiffness) could predict whether or not women taking AI as their adjuvant therapy for breast cancer would develop AIA symptoms. However, our final sample size limited our ability to perform the appropriate statistical models that would allow for prediction of AIA. Despite this setback, our results suggest that increased SW velocity (suggestive of increased stiffness) at baseline in the abductor pollicis longus was associated with a worsening of all three pain and stiffness measures by 6 months. Additionally, an increased velocity in two of six tendons and the median nerve was associated with greater pain at baseline, whereas a slower velocity of the extensor digitorum tendon (suggestive of decreased stiffness) was associated with a greater WOMAC stiffness score. Furthermore, we identified important differences in SW velocity by the image axis and between dominant and non-dominant hands that can inform standardization procedures for SWE image collection in future studies.

At baseline, there were significant differences in SW velocity between dominant and non-dominant sides for four of six tendon sites as well as the median nerve. The greatest difference was observed for the abductor pollicis longus, which had a significantly faster median SW velocity, suggesting stiffer tendons, on the dominant side in the long axis (15.7% difference) than the non-dominant side. The abductor pollicis longus is responsible for facilitating movement and stabilization of the thumb, and the increased velocity in this tendon may be related to high use of the thumb, particularly on the dominant side. The extensor pollicis brevis had a faster median SW velocity on the dominant side than the non-dominant side but in the short axis only (9.1% difference). The flexor digitorum profundus and flexor digitorum superficialis both had significantly slower velocities on the dominant side than the non-dominant side in the short axis only (14.3% and 10.2% slower, respectively). The importance of evaluating these differences in SW velocities by side has been suggested in other studies for different tendons. For example, in athletes with unilateral patellar tendonitis, the more painful side was significantly more stiff than the less painful side (169% difference) and was significantly more stiff than the dominant side patellar tendon of controls (159% difference). However, there was no difference by side within the healthy control group [27]. Siu et al. showed that the Achilles tendon of the nondominant ankle was significantly stiffer in frequent exercisers than in infrequent exercisers, but there was no difference between exercise groups on the dominant side, and there were no within-person differences in stiffness between sides [28]. Couppe’ et al. also observed no difference in SW velocities of the patellar tendon between sides in healthy individuals [29]. A study by Hsiao et al. suggested that aging plays a significant role in the differences in SW velocities of the patellar tendon between sides, with older healthy individuals having a larger difference between sides (10.8% stiffer left side) than younger individuals (6.3% stiffer left side) [30]. Notably, the oldest group in the Hsiao study was on par with the current study. To our knowledge, there are no other studies that directly compare differences in SW velocities between dominant and non-dominant hands in the tendons of the hands and wrists. Whether the percent differences in SW velocities between sides observed in this study represent a clinically meaningful difference for arthralgias is unknown, particularly given that AIA presents bilaterally.

For some tendons (e.g., the Achilles tendon), it has consistently been shown that softer tendons are associated with symptomatic findings; however, the opposite has been observed in the rotator cuff and patellar tendon [19,31]. For the median nerve, faster SW velocities are associated with symptomatic findings, such as carpal tunnel syndrome [18]. Our previous work suggested that women on AI for treatment of their breast cancer that reported pain in their hands and wrists had stiffer tendons than age-matched healthy women [12]. However, we were unable to determine whether stiffer tendons were a result of AI treatment in our previous study. Therefore, here, we sought to determine whether the degree of tendon stiffness changed with AI treatment or remained constant. To our knowledge, this is the first study to record changes in SW velocities of the tendons of hands and wrists over time in breast cancer patients on AI. There was a significant decrease in SW velocities in the long axis (suggesting tendon softening) for three of six tendons, but there was an increased velocity for one of six tendons in the short axis (suggesting tendon stiffening). There was also an increased SW velocity over 6 months for the median nerve in both the long and short axes but a decrease in the short axis on the non-dominant side. Overall, the magnitude of changes was greater in the dominant hand for all anatomical sites. However, absolute changes in velocity were very small (all < 1%) and may not be clinically relevant. Future studies with age-matched controls followed for an equal time are necessary to determine whether these small changes while on AI treatment over 6 months represent clinically meaningful changes in tendon stiffness or are indeed a result of AI treatment.

Several patients had elevated pain and stiffness at baseline quantified by the WOMAC and BCPT-MS questionnaires, prior to AI treatment. Therefore, we next sought to determine whether tendon stiffness at baseline was associated with these measures. There were two of six tendon sites that were significantly stiffer among patients with a BCPT-MS score > 0, and one of six was stiffer with a WOMAC stiffness score > 0. Interestingly, the median nerve had significantly faster velocities at baseline in both axes on the dominant side in women with a BCPT-MS score > 0. This is consistent with other studies noting a relationship between symptomatic findings and a stiffer median nerve [18]. Notably, at baseline, the median (IQR) questionnaire scores were very low: BCPT-MS, 0.83 (0.67–1.0); WOMAC stiffness, 12.5 (12.5–25). There was one person with a BCPT-MS score > 1.5 at baseline, which other studies have used as a cut-off for the development of AIA [24,32]. Additionally, using WOMAC, there was only one person with severe stiffness at baseline (the same person with BCPT-MS score > 1.5), three with moderate stiffness, and five with mild stiffness. Thus, while some women had symptoms present at baseline, only one person had scores equivalent to what is found with AIA. Future studies with a larger sample size should evaluate SW velocities in relationship to established AIA.

There were 14 patients that had both baseline SWE images and paired baseline to 6-month pain and stiffness questionnaire data. Higher SW velocity in the abductor pollicis longus at baseline was associated with a worsening of all three subscales. While the difference in baseline SW velocity only reached statistical significance for the BCPT-MS and WOMAC stiffness scores, there was a 185% higher SW velocity at baseline among women with higher WOMAC pain scores at 6 months for the abductor pollicis longus. While no other studies have demonstrated a relationship between SWE scores in the abductor pollicis longus and the development of AIA, this finding is consistent with a small study showing symptomatic findings on MRI in the abductor pollicis longus in two women that developed AIA [33]. Similarly, in a case study of a woman with AIA, the abductor pollicis longus showed thickening on US, and a diagnosis of tendinopathy was made [34]. In the current study, the only other tendon for which increased SW velocities at baseline were associated with AIA outcomes at 6 months was the flexor digitorum superficialis. The flexor tendons are supported by the transverse carpal ligament, which has been shown to have increased SW velocities with repetitive hand use prior to the onset of symptoms, such as in pianists [35]. Given that the flexor digitorum superficialis flexes both the middle and proximal phalanges, it may be the first to fail and possibly be representative for the hand. Notably, however, there was no difference at baseline in the flexor digitorum superficialis between symptomatic and asymptomatic patients, as measured by any pain or stiffness questionnaire. A 20-point change in the WOMAC pain and stiffness scores is considered the minimally clinically important difference [36]. Here, six of eight participants with worsening scores reached a 20-point increase on the WOMAC stiffness scale, and three of five participants reached it on the WOMAC pain scale. A larger sample size is needed to evaluate the relationship between SW velocities and WOMAC pain and stiffness scores in order to determine clinically relevant differences in SW velocities based on these scales. Future studies should confirm whether SW velocities in the abductor pollicis longus predict AIA outcomes in breast cancer patients.

A critical weakness of our study is a small sample size (*n* = 16) with an even smaller paired baseline-to-post-treatment subset for the SWE component (*n* = 9). However, there were 14 patients with paired baseline SWE images and 6-month questionnaire data that allowed for generating hypotheses regarding the use of SWE as a predictive imaging biomarker of AIA, particularly for the abductor pollicis longus tendon. Given the small sample size, all results should be considered hypothesis-generating; many of the observed changes were small and need to be tested in a larger sample size. Despite the small sample size, we were still able to identify important differences between dominant and non-dominant hands in both the long and short axes that could inform image acquisition for future studies. The use of two separate transducers (9–4 MHz and 12 MHz) is also a limitation [37]. It is possible that some of the observed differences were due to the error introduced with a higher-frequency transducer. However, we adjusted for transducer in the analysis, obtained images in triplicate, and used the entire anatomical image—all of which increase confidence in our results. We were also limited in that the majority of women already had musculoskeletal symptoms (BCPT-MS) and stiffness (WOMAC stiffness) at baseline; however, all but one of these were mild to moderate. We initially projected that 50% of our population would develop AIA based on the BCPT-MS score of ≥1.5 [24]; however, only four of 14 (29%) participants reached that threshold at 6 months. Future studies should select patients with limited or no musculoskeletal symptoms at baseline or select scales with a wider range to better define the changes with AI treatment by SWE. Nonetheless, our prospective study design, including acquisition of the images in triplicate and use of the entire anatomical image to calculate SWE scores, has strengths which contribute an innovative technique to the literature.

## 5. Conclusions

In conclusion, we have shown that (1) it is feasible to recruit women with breast cancer not yet experiencing AI symptoms and to collect an SWE measurement at two timepoints, (2) this method of collecting SWE data is reproducible and that capturing SW velocity measurements of each tendon in triplicate is also feasible, (3) study procedures impose a low patient burden, (4) it is important to capture both dominant and non-dominant sides, and (5) we have refined the code to quantify SWE measurements. Furthermore, our results suggest that the abductor pollicis longus tendon could be an important anatomical site to predict AIA in breast cancer patients using US SWE. Given the small sample size, all findings should be considered as hypothesis-generating. Future studies should evaluate SW velocities related to pain and stiffness outcomes in a larger sample size. Future studies should also consider hand dominance and image acquisition along both axes when reporting clinical outcomes.

## Figures and Tables

**Table 1:** Baseline characteristics in the shear wave elastography cohort (*n* = 16).

Characteristic	Median (IQR) or *n* (%)
Age at enrollment (y)	64.9 (63.5–71.5)
Age at diagnosis (y)	64.4 (63.1–71.2)
Time since diagnosis (months)	4.6 (3.3–6.3)
BMI (kg/m^2^)	25.9 (23.4–33.6)
Right-side dominant	15 (94%) ^a^
Race/ethnicity	
Non-Hispanic white	14 (87.5%)
Hispanic	2 (12.5%)
Definitive breast surgery	
Mastectomy	4 (25.0%)
Lumpectomy	12 (75.0%)
Radiation	
No	6 (37.5%)
Yes	10 (62.5%)
Disease stage	
0	3 (18.8%)
I	11 (68.8%)
II	2 (12.5%)

^a^ Participants were contacted retroactively and asked about handedness. Four participants could not be reached and were classified as right-handed. Abbreviations: BMI: body mass index.

**Table 2:** (a) Baseline shear wave elastography velocity (m/s) for long axis: mean ± SD ^a^. (b) Baseline shear wave elastography velocity (m/s) for short axis: mean ± SD ^a^.

(**a**)			
**Image Location**	**Dominant Side**	**Non-Dominant Side**	***p*-Value**
Abductor pollicis longus	5.59 ± 2.46	4.71 ± 2.14	0.020
Extensor carpi ulnaris	4.76 ± 1.67	4.64 ± 1.64	0.568
Extensor digitorum tendon	5.55 ± 1.51	5.93 ± 2.22	0.157
Extensor pollicis brevis	4.00 ± 1.21	4.11 ± 1.71	0.684
Flexor digitorum profundus	6.77 ± 2.23	6.45 ± 2.77	0.389
Flexor digitorum superficialis	6.09 ± 1.59	6.47 ± 1.99	0.162
Median nerve	5.56 ± 2.08	6.18 ± 1.70	0.020
(**b**)			
**Image Location**	**Dominant Side**	**Non-Dominant Side**	***p*-Value**
Abductor pollicis longus	4.47 ± 1.07	4.41 ± 0.86	0.728
Extensor carpi ulnaris	4.42 ± 0.62	4.33 ± 0.69	0.342
Extensor digitorum tendon	5.03 ± 0.99	4.91 ± 1.29	0.548
Extensor pollicis brevis	4.72 ± 0.71	4.29 ± 0.95	0.007
Flexor digitorum profundus	4.53 ± 0.67	5.18 ± 1.30	<0.001
Flexor digitorum superficialis	4.51 ± 0.82	4.97 ± 1.43	0.045
Median nerve	5.02 ± 1.31	5.21 ± 1.49	0.413

^a^ Mixed-effects model adjusted for the transducer, clustered on the patient (no adjustments for multiple comparisons).

**Table 3:** Shearwave elastography change in velocity (m/s) over time (6 months): beta-coefficient (*p*-value) ^a^.

Image Location	Long Axis Dominant Side	Short Axis Dominant Side	Long Axis Non-Dominant Side	Short Axis Non-Dominant Side
Abductor pollicis longus	−0.024 (0.027)	0.002 (0.701)	0.002 (0.849)	−0.001 (0.797) ^b^
Extensor carpi ulnaris	−0.033 (0.000)	0.006 (0.040) ^b^	−0.000 (0.996)	0.002 (0.570) ^b^
Extensor digitorum tendon	0.002 (0.817)	−0.006 (0.257)	0.004 (0.698)	−0.006 (0.363) ^b^
Extensor pollicis brevis	0.006 (0.485)	0.007 (0.148) ^b^	0.007 (0.507)	0.005 (0.458)
Flexor digitorum profundus	0.022 (0.084)	0.003 (0.329) ^b^	0.012 (0.453)	−0.004 (0.351)
Flexor digitorum superficialis	−0.024 (0.014)	0.007 (0.138) ^b^	−0.020 (0.038) ^b^	−0.007 (0.332)
Median nerve	0.031 (0.002)	0.018 (0.009)	−0.021 (0.019)	0.016 (0.113)

^a^ Mixed-effects model with time (date) as a continuous variable, adjusted for baseline velocity (mean of three measures) and the transducer, clustered on the patient (no adjustments for multiple comparisons); ^b^ The random-effect constant could not be reasonably estimated. Significance was unchanged in the sensitivity analyses.

## Data Availability

The data presented in this study are available on request from the corresponding author. The data are not publicly available due to containing information that could compromise the privacy of research participants.

## References

[1] Park S.-H., Knobf M.T., Sutton K.M. (2012). Etiology, Assessment, and Management of Aromatase Inhibitor-Related Musculoskeletal Symptoms. Clin. J. Oncol. Nurs..

[2] Sestak I., Sapunar F., Cuzick J. (2009). Aromatase Inhibitor–Induced Carpal Tunnel Syndrome: Results From the ATAC Trial. J. Clin. Oncol..

[3] Spagnolo F., Sestak I., Howell A., Forbes J.F., Cuzick J. (2016). Anastrozole-Induced Carpal Tunnel Syndrome: Results From the International Breast Cancer Intervention Study II Prevention Trial. J. Clin. Oncol..

[4] Dizdar O., Özçakar L., Malas F.Ü., Harputluoglu H., Bulut N., Aksoy S., Ozisik Y., Altundag K. (2009). Sonographic and Electrodiagnostic Evaluations in Patients with Aromatase Inhibitor–Related Arthralgia. J. Clin. Oncol..

[5] Morales L., Pans S., Verschueren K., Van Calster B., Paridaens R., Westhovens R., Timmerman D., De Smet L., Vergote I., Christiaens M.-R. (2008). Prospective Study to Assess Short-Term Intra-Articular and Tenosynovial Changes in the Aromatase Inhibitor–Associated Arthralgia Syndrome. J. Clin. Oncol..

[6] Presant C.A., Bosserman L., Young T., Vakil M., Horns R., Upadhyaya G., Ebrahimi B., Yeon C., Howard F. (2007). Aromatase Inhibitor–Associated Arthralgia and/or Bone Pain: Frequency and Characterization in Non–Clinical Trial Patients. Clin. Breast Cancer.

[7] Gaillard S., Stearns V. (2011). Aromatase inhibitor-associated bone and musculoskeletal effects: New evidence defining etiology and strategies for management. Breast Cancer Res..

[8] Henry N.L., Giles J.T., Ang D., Mohan M., Dadabhoy D., Robarge J., Hayden J., Lemler S., Shahverdi K., Powers P. (2007). Prospective characterization of musculoskeletal symptoms in early stage breast cancer patients treated with aromatase inhibitors. Breast Cancer Res. Treat..

[9] Howell A. (2005). Results of the ATAC (Arimidex, Tamoxifen, Alone or in Combination) trial after completion of 5 years’ adjuvant treatment for breast cancer. Lancet.

[10] Castel L.D., Hartmann K.E., Mayer I.A., Saville B.R., Alvarez J., Boomershine C.S., Abramson V.G., Chakravarthy A.B., Friedman D.L., Cella D.F. (2013). Time course of arthralgia among women initiating aromatase inhibitor therapy and a postmenopausal comparison group in a prospective cohort. Cancer.

[11] Goss P.E., Ingle J.N., Pritchard K.I., Robert N.J., Muss H., Gralow J., Gelmon K., Whelan T., Strasser-Weippl K., Rubin S. (2016). Extending Aromatase-Inhibitor Adjuvant Therapy to 10 Years. N. Engl. J. Med..

[12] Martinez J.A., Taljanovic M.S., Witte R.S., Zuniga A.A.N., Wertheim B.C., Kwoh C.K., Goldstein B.A., Roe D.J., Chalasani P. (2021). Shear wave elastography detects novel imaging biomarkers of aromatase inhibitor–induced joint pain: A pilot study. J. Ultrason..

[13] Shanmugam V.K., McCloskey J., Elston B., Allison S.J., Eng-Wong J. (2011). The CIRAS study: A case control study to define the clinical, immunologic, and radiographic features of aromatase inhibitor-induced musculoskeletal symptoms. Breast Cancer Res. Treat..

[14] Henry N.L., Jacobson J., Banerjee M., Hayden J., Smerage J.B., Van Poznak C., Storniolo A.M., Stearns V., Hayes D.F. (2010). A prospective study of aromatase inhibitor-associated musculoskeletal symptoms and abnormalities on serial high-resolution wrist ultrasonography. Cancer.

[15] Sarvazyan A.P., Rudenko O., Swanson S.D., Fowlkes J., Emelianov S. (1998). Shear wave elasticity imaging: A new ultrasonic technology of medical diagnostics. Ultrasound Med. Biol..

[16] Taljanovic M.S., Gimber L.H., Becker G.W., Latt L.D., Klauser A.S., Melville D.M., Gao L., Witte R.S. (2017). Shear-Wave Elastography: Basic Physics and Musculoskeletal Applications. RadioGraphics.

[17] Turkay R., Inci E., Aydeniz B., Vural M. (2017). Shear wave elastography findings of de Quervain tenosynovitis. Eur. J. Radiol..

[18] Giambini H., An K.-N. (2021). Ultrasound Elastography for Hand Soft Tissue Assessment. Hand Clin..

[19] Breda S.J., Van Der Vlist A., De Vos R.-J., Krestin G.P., Oei E.H.G. (2020). The association between patellar tendon stiffness measured with shear-wave elastography and patellar tendinopathy—A case-control study. Eur. Radiol..

[20] Hou S.W., Merkle A.N., Babb J., McCabe R., Gyftopoulos S., Adler R.S. (2016). Shear Wave Ultrasound Elastographic Evaluation of the Rotator Cuff Tendon. J. Ultrasound Med..

[21] Pan W., Zhou J., Lin Y., Zhang Z., Wang Y. (2021). Elasticity of the Achilles Tendon in Individuals with and without Plantar Fasciitis: A Shear Wave Elastography Study. Front. Physiol..

[22] Alfuraih A.M., Tan A.L., O’Connor P., Emery P., Wakefield R.J. (2020). Muscle stiffness in rheumatoid arthritis is not altered or associated with muscle weakness: A shear wave elastography study. Mod. Rheumatol..

[23] Stanton A.L., Bernaards C.A., Ganz P.A. (2005). The BCPT Symptom Scales: A Measure of Physical Symptoms for Women Diagnosed with or at Risk for Breast Cancer. J. Natl. Cancer Inst..

[24] Swenson K.K., Nissen M.J., Henly S., Maybon L., Pupkes J., Zwicky K., Tsai M.L., Shapiro A.C. (2013). Identification of Tools to Measure Changes in Musculoskeletal Symptoms and Physical Functioning in Women with Breast Cancer Receiving Aromatase Inhibitors. Oncol. Nurs. Forum.

[25] Bellamy N., Buchanan W.W., Goldsmith C.H., Campbell J., Stitt L.W. (1988). Validation study of WOMAC: A health status instrument for measuring clinically important patient relevant outcomes to antirheumatic drug therapy in patients with osteoarthritis of the hip or knee. J. Rheumatol..

[26] Chen L., Lin C.-C., Huang T.-W., Kuan Y.-C., Huang Y.-H., Chen H.-C., Kao C.-Y., Su C.-M., Tam K.-W. (2017). Effect of acupuncture on aromatase inhibitor-induced arthralgia in patients with breast cancer: A meta-analysis of randomized controlled trials. Breast.

[27] Zhang Z.J., Ng G.Y.-F., Lee W.C., Fu A. (2014). Changes in Morphological and Elastic Properties of Patellar Tendon in Athletes with Unilateral Patellar Tendinopathy and Their Relationships with Pain and Functional Disability. PLoS ONE.

[28] Siu W.-L., Chan C.-H., Lam C.-H., Lee C.-M., Ying M. (2016). Sonographic evaluation of the effect of long-term exercise on Achilles tendon stiffness using shear wave elastography. J. Sci. Med. Sport.

[29] Couppé C., Kongsgaard M., Aagaard P., Hansen P., Bojsen-Moller J., Kjaer M., Magnusson S.P. (2008). Habitual loading results in tendon hypertrophy and increased stiffness of the human patellar tendon. J. Appl. Physiol..

[30] Hsiao M.-Y., Chen Y.-C., Lin C.-Y., Chen W.-S., Wang T.-G. (2015). Reduced Patellar Tendon Elasticity with Aging: In Vivo Assessment by Shear Wave Elastography. Ultrasound Med. Biol..

[31] Washburn N., Onishi K., Wang J.H.-C. (2018). Ultrasound elastography and ultrasound tissue characterisation for tendon evaluation. J. Orthop. Transl..

[32] Shapiro A.C., Adlis S.A., Robien K., Kirstein M.N., Liang S., Richter S.A., Lerner R.E. (2016). Randomized, blinded trial of vitamin D3 for treating aromatase inhibitor-associated musculoskeletal symptoms (AIMSS). Breast Cancer Res. Treat..

[33] Singer O., Cigler T., Moore A.B., Levine A.B., Hentel K., Belfi L., Do H.T., Mandl L.A. (2012). Defining the aromatase inhibitor musculoskeletal syndrome: A prospective study. Arthritis Care Res..

[34] Martens H.A., Schroder C.P., van der Eerden P.J.M., Willemse P.H.B., Posthumus M.D. (2007). Severe disabling tendinopathy caused by anastrazole. Rheumatology.

[35] Mhanna C., Marquardt T.L., Li Z.-M. (2016). Adaptation of the Transverse Carpal Ligament Associated with Repetitive Hand Use in Pianists. PLoS ONE.

[36] Tubach F., Ravaud P., Beaton D., Boers M., Bombardier C., Felson D.T., Van Der Heijde D., Wells G., Dougados M. (2007). Minimal clinically important improvement and patient acceptable symptom state for subjective outcome measures in rheumatic disorders. J. Rheumatol..

[37] O’Hara S., Zelesco M., Rocke K., Stevenson G., Sun Z. (2019). Reliability Indicators for 2-Dimensional Shear Wave Elastography. J. Ultrasound Med..

